# Team VA Video Connect (VVC) to optimize mobility and physical activity in post-hospital discharge older veterans: baseline assessment

**DOI:** 10.1186/s12877-021-02454-w

**Published:** 2021-09-22

**Authors:** Neil B. Alexander, Kristin Phillips, Joleen Wagner-Felkey, Chiao-Li Chan, Robert Hogikyan, Alexandra Sciaky, Christine Cigolle

**Affiliations:** 1 VA Ann Arbor Healthcare System Geriatric Research, Education and Clinical Center (GRECC), 2215 Fuller Road, MI 48105 Ann Arbor, USA; 2 Division of Geriatric and Palliative Medicine, Department of Internal Medicine, Ann Arbor, USA; 3 Department of Family Medicine, University of Michigan, Ann Arbor, USA

## Abstract

**Background:**

Telehealth is increasingly used for rehabilitation and exercise but few studies include older adult participants with comorbidities and impairment, particularly cognitive. Using Veterans Administration Video Connect (VVC), the aim of the present study is to present the screening, recruitment, baseline assessment, and initial telehealth utilization of post-hospital discharge Veterans in a VVC home-telehealth based program to enhance mobility and physical activity.

**Methods:**

Older adult Veterans (*n* = 45, mean age 73), recently discharged from the hospital with physical therapy goals, were VVC-assessed in self-report and performance-based measures, using test adaptations as necessary, by a clinical pharmacy specialist and social worker team.

**Results:**

Basic and instrumental ADL disabilities were common as were low mobility (Short Portable Performance Battery) and physical activity levels (measured by actigraphy). Half had Montreal Cognitive Assessment (MoCA) scores in the mild cognitive impairment range (< 24). Over 2/3 of the participants used VA-supplied tablets. While half of the Veterans were fully successful in VVC, 1/3 of these and an additional group with at least one failed connection requested in-person visits for assistance. One-quarter had no VVC success and sought help for tablet troubleshooting, and half of these eventually “gave up” trying to connect; difficulty with using the computer and physical impairment (particularly dexterity) were described prominently in this group. On the other hand, Veterans with at least mild cognitive impairment (based on MoCA scores) were present in all connectivity groups and most of these used caregiver support to facilitate VVC.

**Conclusions:**

Disabled older post-hospital discharged Veterans with physical therapy goals can be VVC-assessed and enrolled into a mobility/physical activity intervention. A substantial proportion required technical support, including in-person support for many. Yet, VVC seems feasible in those with mild cognitive impairment, assuming the presence of an able caregiver. Modifications of assessment tools were needed for the VVC interface, and while appearing feasible, will require further study.

**Trial registration:**

ClinicalTrials.gov NCT 04045054 05/08/2019.

## Introduction

Older adults (aged ≥ 65) decline in functional status leading up to and during an acute hospitalization and are at high risk for disability, not just related to the initial acute illness, but also to the accumulation of deficits termed post-hospital syndrome [1]. Many are deconditioned at hospital discharge to a functional mobility and physical activity level below that of their baseline pre-admission level [2, 3]. Many of the successful post-hospital discharge transitional care programs appropriately focus on medical issues, using a nurse care manager, or on specific disease management (such as heart failure), but do not necessarily focus on physical function, and optimizing mobility and physical activity in particular [4].

Hospitalized patients commonly are discharged prior to reaching their full mobility potential, despite initiation of physical therapy during their acute hospitalization. Some are discharged to post-acute care, while others go home to receive outpatient or home therapy or often no services. For those sent home with plans for therapy, the rehabilitation programs tend to include fewer and shorter duration visits, partly due to reimbursement limitations [5]. Patients living in rural areas may not receive full access to these services due to the distances patients or therapists must travel and the fewer therapists available. There is great interest in improving transitions between the hospital and home, and enhancements can include increased patient education and provider-patient support [6]. 

One solution to improving these transitions is to provide support via telehealth. Veterans can conduct remote video visits with healthcare providers via Veterans Administration Video Connect (VVC). Geriatric Research Education and Clinical Center (GRECC) Connect has been successful in linking older Veterans and care providers using Clinical Video Telehealth (CVT), in which the Veteran is supported by a Community Based Outpatient Clinic (CBOC) [7]; GRECC Connect is in the process of adding a VVC model. GRECC Connect provides support across a range of geriatric problems, but is not specifically focused on optimizing mobility and physical activity.

Yet, few telehealth studies address care for older adults, particularly those with comorbidities and cognitive impairment, or the importance of caregiver participation. While telehealth interventions have been provided for persons with cognitive impairment, a recent review of video telehealth programs targeting persons with dementia and their caregivers found that the majority of telehealth interventions targeted caregivers and there was a relative lack of information on patient outcomes [8]. Nevertheless, video telehealth in the cognitively impaired can be successful, with one recent study demonstrating successful VVC call connection in older Veterans with mild cognitive impairment as long as there was a care partner present [9], a strategy also used in the present study. Furthermore, telehealth-based exercise has been used with a number of modalities, including web-based, mobile applications, text messaging, and telephone interventions. Interventions which lead to increased physical activity and reduced sedentary behavior use many of these same telehealth modalities, but tend to exclude the older, comorbid and cognitively impaired, regardless of caregiver participation [10].

Building on the limits of the studies above, the aim of the present study was to deliver a post-hospital discharge VVC home-telehealth based program to enhance mobility and physical activity, that was lifestyle-oriented, caregiver-supported (as needed) and longer term (6 months). An additional objective was to explore the feasibility of modifications utilized to perform VVC versions of standard (unabridged) cognitive and mobility assessments, typically done in person. Patients targeted were those evaluated by physical therapy prior to hospital discharge with rehabilitation goals and the potential to improve their mobility. Given concerns for ongoing medical and social issues that might interfere with the program, including the presence of cognitive impairment, a multidisciplinary team (including a pharmacist, social worker, and physician, as needed) completed baseline assessments and initial interventions to facilitate the trainer-provided, customized mobility and physical activity enhancement program. The present report focuses on the screening, recruitment, baseline assessment, and initial telehealth utilization during the period of enrollment and baseline testing. The mobility and physical activity intervention and outcomes will be reported in a subsequent manuscript.

## Methods

### Inclusion and exclusion criteria

Older patients on hospital discharge were recruited to participate in a 6-month intervention designed to improve safe mobility and physical activity. Key inclusion criteria were: (1) age 50 or older, (2) recent acute hospital discharge, and (3) a physical therapy evaluation during the hospitalization with stated rehabilitation goals on discharge. The purpose of the latter criteria was to ensure that the participant had rehabilitation goals that should be targeted. These goals included improvements in bed and chair transfers, safe ambulation with or without an assistive device, increased endurance, pain reduction, and initiation of a home exercise program. Duration of treatment to achieve these goals was not specified. Of the 45 participants reported on during this baseline period, sixteen were enrolled in physical therapy post-discharge, nine in-home, and seven outpatient, with no clear differences in goals specified.

Potential participants with known cognitive impairment and support by a caregiver were recruited as a dyad. Participants were initially required to have an established primary care provider at the VA medical center where the study was based, although this was later revised to require a primary care provider at any statewide VA medical center.

Veterans who were dependent on key highly focused interventions and/or were not likely to benefit from a 6 month walking-oriented intervention were excluded, i.e. those who: (1) required highly specialized equipment (e.g., spinal cord injury, leg amputation, wound [Vacuum Assisted Closure], heavy leg boot), (2) had an active mental health condition or active substance use that might directly interfere with participation; (3) required ambulation or transfer assistance beyond an assistive walking device, i.e. requiring strict bedrest, use of a wheelchair, or dependency on another person for safe mobility; (4) had life expectancy or planned state residency < 6 months; (5) had other severe and/or unresolved medical conditions or ongoing complicating treatments (e.g. BMI > 45, constant home oxygen use > 2 L/minute, > 3 hospital admissions in the last 6 months, ongoing active chemotherapy with toxicity); (6) had severe sensory (e.g. blindness), speech (e.g. tracheostomy) or cognitive loss that could not be caregiver compensated; (7) required a caregiver or family support but the support was not dependably available or was complicated by family dynamics; or (8) had a planned discharge to post-acute or long term care setting. Those enrolled in potentially parallel programs that might duplicate aspects of the program were also excluded, such as those with planned cardiac rehabilitation participation as well as care by VA Home Based Primary Care.

### Recruitment

Study team members obtained access to inpatient physical therapy consult rosters in the Veterans Health Information System Technology Architecture (VistA). These lists were screened approximately three times per week to identify potential participants based on the inclusion and exclusion criteria. While the protocol was initially designed to recruit participants prior to discharge, uncertainty, complexity, and changing nature of discharge plans, inability to consistently contact the participant prior to discharge, and participant desire to go home prior to consent required other recruitment approaches. These approaches included a telephone call, meeting at a follow-up outpatient appointment, or an introduction letter with study team phone follow-up. Of the 45 participants reported here, 11 ultimately completed study consent as inpatients, and the other 34 completed consent post-discharge.

Nevertheless, delays in post-discharge enrollment for most were modest, with 2/3 enrolled within 1–3 weeks post discharge. Approximately 1/3 (n = 14) were enrolled over 3 weeks post discharge. Overall, the mean (SD) days from discharge to enrollment were 17 (14) days and the median [25th, 75th percentile] days were 12 [7,22]. A median split analysis comparing early (≤ 12 days) versus late (> 12 days) enrollment showed no differences in various functional measures, Montreal Cognitive Assessment (MoCA) scores, or activity monitor metrics.

Data reflect participants recruited from late 2017 to early 2020 (ending immediately prior to the COVID outbreak).

### VVC equipment

A compatible personal home device was prioritized, anticipating faster adoption of VVC and greater ease of use. If the participant did not have access to a compatible home device, a tablet device (iPad) was mailed directly to the participant. To acquire the tablet, a study team member entered a consult to the local Telehealth Telecommunications Technician, who then put in an order with the VA Denver Acquisitions and Logistics Center (DALC). The tablets are encrypted and meet VA standards for secure transmission. The tablets could only be used for VVC connection and VA applications were locked down by the DALC to only be used for those purposes. Supported by the local VA telehealth coordinator and technicians as well as the national telehealth help desk, experienced study team members worked with participants/caregivers to support tablet and other device use and to schedule appointments.

### VVC utilization groups

We identified four groups based on full VVC connection success (versus at least one failed VVC or no VVC success), and any in-person (participant-staff) contact: group 1 (full VVC success, no in-person); group 2 (full VVC success, at least one in-person).; group 3 (at least one failed VVC, at least one in-person); and group 4 (no VVC success, at least one in person).

### Team VVC assessments

A team was assembled to address the anticipated medical and psychosocial support needs of a recently discharged Veteran cohort, both of which might interfere with optimal provision of a mobility/physical activity intervention, and that might not be addressed easily and in a timely manner by an outpatient primary care team. This team consisted of a clinician (in this case a clinical pharmacy specialist) who could screen medical issues and seek consultation of a geriatric medicine physician, and a social worker. This model was designed to leverage, and not duplicate, present available home health and outpatient services (especially therapists). Note that the use of a clinical pharmacy specialist also provided an opportunity to address safe medication use, a particularly important issue post-hospital discharge that would be critical for optimal medical care but perhaps not critical to conduct a mobility/physical activity program.

For the eventual intervention, an experienced trainer coached the participant to consolidate the rehabilitation goals (i.e. encourage the participant to follow home exercise instructions given), while adding a safe walking program (to be described in a subsequent intervention paper). A major focus was to link the rehabilitation goals and walking program to everyday lifestyle activities.

The pharmacy specialist and social worker met with the participant and/or caregiver for an initial visit as soon as possible after enrollment, and after the baseline activity monitor was received back from the participant. The visit was most often conducted via VVC, although at times due to connectivity or other issues, some assessments were done via telephone or in person. When possible and applicable, there was an attempt to coordinate the VVC visit in the presence of the participant’s home health physical therapist. This was rarely helpful, given delays in onset of home health services, scheduling conflicts, and the urgency to complete the baseline assessment. Medication review and social work assessments were conducted at the initial visit, along with other assessments and gathering of any demographic information not available via chart review (race, education, marital status, residence/living arrangements, rehabilitation goals). Assessments (see below) were completed usually during 1 session and within 1–2 weeks of enrollment; for the late enrollees noted above (n = 14), the mean (SD) completion of enrollment was delayed to 4.8 (3.8) weeks post-discharge.

For the reported functional data, a score of one was given for: (1) each basic Activities of Daily Living (ADL) task item the participant was able to perform without help (0–6); 2) each instrumental ADL that the participant was able to perform without help (0–8); (3) each Rosow-Breslau item (e.g. walking stairs) that the participant was unable to perform (0–3); and (4) each Nagi item that the participant had difficulty with (e.g. lifting or carrying weights) (0–5) (based on [11]). Caregiver burden was assessed using the 4 item Zarit Burden Interview Screening [12] (score range 0–16, with ≥ 8 considered high burden).

#### Comprehensive medication review

Prior to interview with the participant, the chart was reviewed by the team clinical pharmacy specialist to gather medication orders and other pertinent medication information and history. During the interview, the participant was asked who was responsible for the management of medications, then either the participant or caregiver was asked to gather the medication bottles in the home or a current medication list and show the medications on camera. The participant and/or caregiver was also asked to describe the medication management system being used (e.g. pillbox). Each medication was then reviewed, noting whether the participant followed the directions on administration as noted in the medical record. Medication adherence was assessed by patient/caregiver report and refill history. Participant medication-related questions/concerns were addressed, and medication discrepancies were reconciled with the participant and/or in the medical record. When applicable, potentially inappropriate medications and deprescribing opportunities were identified in final documentation, and the primary care provider was alerted with recommendations.

#### Social work assessment

The team social worker reviewed each participant for eligibility for financial and home health aide benefits. A key feature was identifying needs in a complex older adult population and initiating appropriate consults or education regarding community resources.

#### Montreal Cognitive Assessment (MoCA) administration

MoCA [13] scores from the medical chart were noted if administered as part of geriatrics, neurology, or neuropsychological evaluations in the past 2 months (n = 9) or collected at outpatient face to face visits [n = 14). Based on studies suggesting the MoCA could be reliably administered by remote videoconference [14], the MoCA was also collected during a VVC visit (n = 18). Modifications for VVC-MoCA included holding the visuospatial/executive portion of the assessment up to the camera such that the participant could see and then complete the Trails test verbally. The cube and clock were drawn on a paper at home by the participant, then held up to the camera for a team member to review. For the attention section where the participant typically was asked to tap a hand on the desk when the letter A was said, the participant was asked to raise a hand in view of the camera. Note that there was no difference between in- person and VVC MoCA scores (both mean 24) but the chart-based MoCA scores, possibly collected due to concerns for cognitive impairment, were lower (mean 20) but this difference did not reach statistical significance.

#### Measures of mobility and physical activity- use of the Short Portable Performance Battery (SPPB) and actigraphy (ActivPal)

 The goal was to pilot these tools, as much as possible, using the VVC interface. Used as a measure of mobility, the SPPB assesses standing balance, multiple chair stands and gait speed and predicts important outcomes such as subsequent disability [15]. The activPAL has been used in a variety of settings, not just for step estimation but for non-sedentary behaviors such as time standing [16].

SPPB was performed via VVC, and as needed, while a caregiver was present in the home. Modifications of the SPPB for video telehealth included: (1) asking the participant to prop the tablet or home device on a table in order to be seen head to toe; (2) asking the participant to approximate a 10 foot distance for testing gait speed; and (3) asking the participant to verbalize “start” and “stop” at the beginning and end of the 10 foot walk in case of video/audio delay. For participants with difficulty measuring the 10 foot distance accurately, the participants were asked to estimate the distance using three large steps (since gait speed was anticipated to be slow and each person was to be used as their own control for study outcomes).

Participants wore a physical activity monitor (activPAL3TM, PAL Technologies Ltd., Glasgow, UK) affixed with an adhesive to the mid-thigh and therefore worn (with data collecting) 24 h per day. Participants had the activity monitor affixed in person initially although eventually, the activity monitor was mailed to the participant with illustrated instructions to affix properly, and to be returned in a pre-paid mailer. Participants were instructed to wear the ActivPal for at least one week and continue their usual daily activities. If the participant somehow added additional days of wear, only the seven days following the first significant wear day were analyzed. Data were processed using a MatLab protocol, deriving standard ActivPal metrics. Number of steps per day and percent of the day engaged in sedentary activity, as per ActivPAL standard analytics, was determined. Of the 41 who wore ActivPal at baseline, 6 had unusable data (due to a technical issue or early removal of the ActivPal) and 3 did not return the ActiPal, leaving data available on n = 32.

#### Statistical comparisons

Continuous variables were compared using independent sample t-tests or one-way ANOVA, and categorical variables were compared using chi-square test (Fisher’s exact for small cells), with statistical significance at p < 0.05.

## Results

### Recruitment flow

**Fig. 1:**
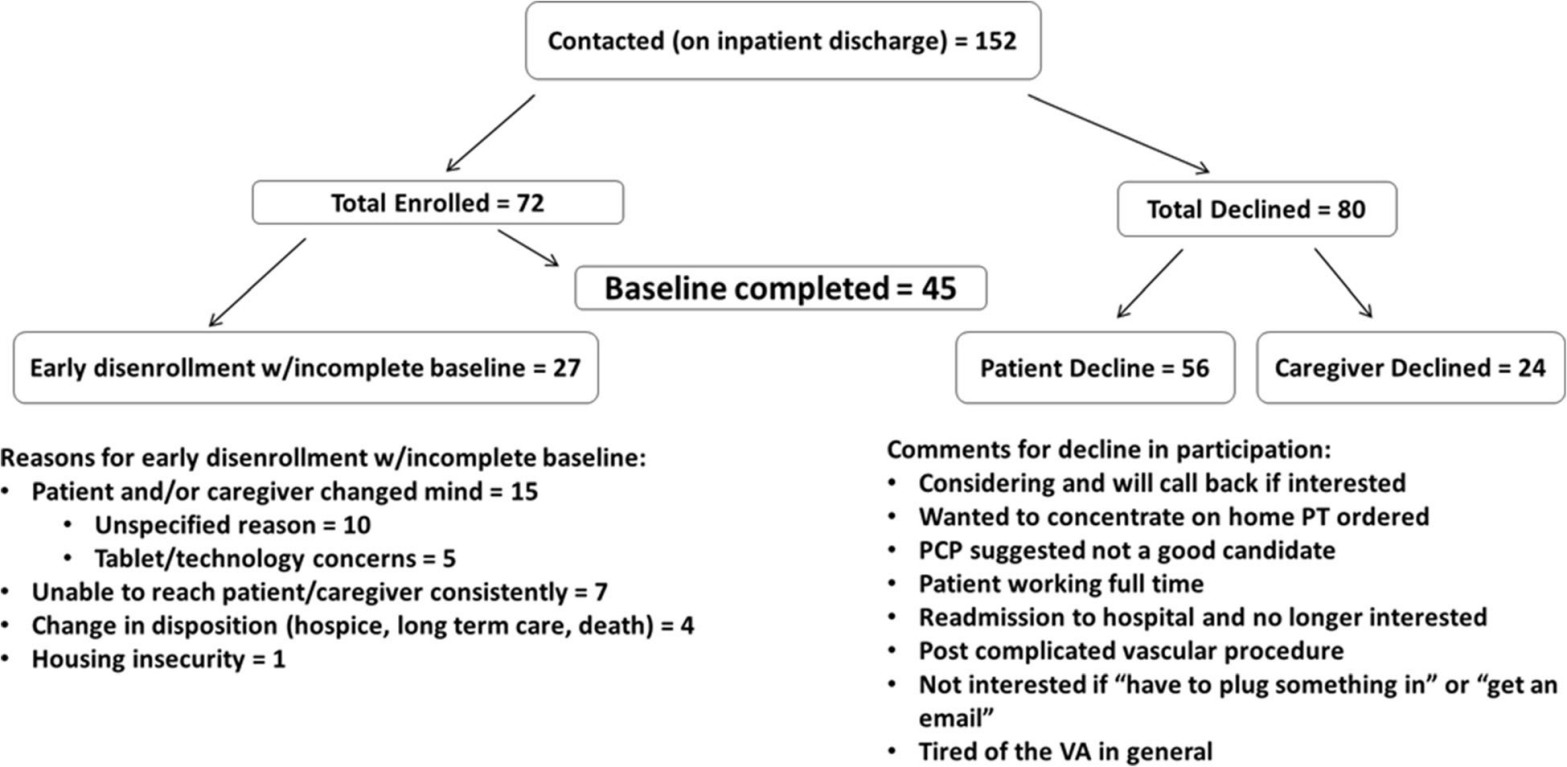
Participant recruitment flow chart.

Of participants considered eligible (n = 152), 80 declined, 56/80 (70 %) by the patient, and 24/80 (30 %) by the caregiver (See Fig. 1). Of the 72 patients enrolled, 27 disenrolled early and had incomplete baseline testing, of which the majority (15/27) changed their minds about participation, thus leaving 45 participants for the present analysis. Connectivity/communication issues were present, given that 5/15 who changed their minds cited tablet/technology concerns, and 7 of the 27 were unable to be reached consistently. The limited data available showed no significant difference between the 27 early disenrollees and the 45 analyzed in basic demographics (e.g. age and marital status).

### Participant characteristics

Mean participant age was 72.9 years and the majority were male, white, married, and educated ≥ 12 years (See Table 1). Self-reported ADL score (mean 4.8 out of 6) indicated disability in approximately one basic ADL. The IADL score (mean 5.6 out of 8) indicated disability in at least 2 IADLs. Similarly, approximately two each of self reported mobility Nagi and Rosow-Breslau items were rated as impaired. Zarit scale caregiver burden was modest. A wide range of principal discharge diagnoses, from both medical and surgical units, included a high percentage of orthopedic/musculoskeletal (40 %) diagnoses (See Table 2).

**Table 1:** Baseline characteristics (*n* = 45 unless otherwise stated)

Gender (% male)	98 %
Race (% white)	96 %
Marital status (% married)	73 %
Education (% ≥ 12 years)	90 %
Depression (PHQ-2) screen (% negative)	91 %
	Mean (SD) [Range]
Age (years)	72.9 (7.5) [51–93]
Montreal Cognitive Assessment (MoCA, *n* = 41)	23.1 (4.3) [11–29]
Zarit Caregiver Burden (*n* = 25)	2.7 (3.4) [0–14]
***Self-reported functional disability***	
Basic ADLs (*n* = 44)	4.8 (1.7) [1-6]
Instrumental ADLs (*n* = 44)	5.6 (2.6) [0–8]
Nagi (*n* = 41)	2.1 (1.3) [0–5]
Rosow-Breslau (*n* = 41)	1.6 (1.0) [0–3]
***Clinical Pharmacy Assessments (n = 41)***	
Mean (SD) medications	11 (4.3)
Medication management	
Patient only n (%)	21 (51 %)
Caregiver only n (%)	12 (29 %)
Both n (%)	8 (20 %)
Participants with discrepancies n (%)	41 (100 %)
Mean (SD) discrepancies	3.8 (2.7)
Participants with potential inappropriate meds n (%)	12 (29 %)
Total # inappropriate meds	15
Total # medication-related problems identified per participant	1.6
***Mobility performance (n = 41)***	
Short Portable Performance Battery (SPPB)	4.9 (3.3) [0–11]
Balance subscale	2.4 (1.3) [0–4]
Chair stand subscale	1.0 (1.2) [0–4]
Gait speed subscale	1.6 (1.1) [0–4]
***Actigraphy (activPAL) (n = 32)***	
% of day sedentary (by hour)	84 (10) [55–97]
Total # steps	2334 (1750) [122–7568]

**Table 2:** Principal Diagnosis on Hospital Discharge (*n* = 45)

Diagnosis area	# participants	% of group
Orthopedic/Musculoskeletal	18	40
*Knee replacement*	*7*	*16*
*Hip replacement*	*3*	*7*
Vascular/Pulmonary	10	22
Gastrointestinal/Renal/Urological	8	18
Infection	7	16
Metabolic	2	4

### Caregiver participation

Thirty of the 45 participants (67 %) participated with a caregiver. Most of the caregivers were spouses (*n* = 23, 77 %), and the remainder an adult child (*n* = 5, 17 %) or sibling (*n* = 2, 7 %). At the beginning of the study, caregivers were required. This requirement was later waived as it became clear that many, even cognitively impaired participants, did not have or did not want caregiver support (see MoCA assessment below). Thus, of the last 16 participants recruited after the requirement change (16/45, 36 %), only one was recruited with a caregiver. In terms of cognitive function (mean [SD] MoCA score), the score of the cohort recruited pre-caregiver requirement change (22.4 [4.4]) tended to be lower than the cohort post-caregiver requirement change (24.7 [3.8]), but the difference was not statistically significant.

### MoCA assessment

Of the 41 participants with cognitive testing available, 21 (51 %) had baseline MoCA scores in the impaired range, based on < 22 as the most accurate MoCA cut score to identify a clinically relevant level of impairment and < 24 to identify milder cognitive impairment for a post-acute hospitalization Veteran cohort [17]. 10 had mild cognitive impairment (MoCA 22–23) and 11 had MoCA < 22.

### VVC use and contributors to VVC success during enrollment and baseline testing

Of the 45 participants, 31 (69 %) used the VA tablet and 14 used a home device. Defining “VVC success” as both participant and assessor able to use the audio and visual feed meaningfully (i.e. both parties able to use both modalities to communicate), about half of the Veterans were fully successful in the VVC interactions (Group 1 and 2, n = 23) (see Table 3). 1/3 of these Veterans (Group 2, n = 8) requested additional in-person visits due to a preference for in-person contact. Group 3 had at least one failed connection (n = 11) and used in-person visits to address connectivity and device use. About one-quarter had no VVC success (Group 4, n = 11) and sought help for tablet troubleshooting; half of these (n = 6) eventually “gave up” trying to connect and defaulted to telephone contact. Difficulty with using the computer and physical impairments (particularly dexterity) were more prominent in Group 4. About half of each of Group 1 and Group 4 used their own devices, the former succeeded in connecting, the latter not succeeding. Veterans with at least mild cognitive impairment (based on MoCA scores) were present in all groups and most of these used caregiver support to facilitate VVC.

**Table 3:** Contributors to VVC Success During Enrollment and Baseline Assessment (*n* = 45)

*VVC Connection Success* *(≥ 1 connection)*	Group 1 Fully Successful *n* = 15	Group 2 Fully Successful *n* = 8	Group 3 ≥ 1 Failed VVC *n* = 11	Group 4 No VVC Success *n* = 11
***Use of in-person contact***	No in-person	≥ 1 in-person	≥ 1 in-person	≥ 1 in-person
***Cognitive level (MoCA)***				
No impairment ≥24	5 (0 w/CG)*	3 (3 w/CG)	5 (5 w/CG)	7 (3 w/CG)
Mild Impairment (22–23)	5 (4 w/CG)	3 (3 w/CG)	2 (1 w/CG)	0
Clinical Impairment (< 22)	1 (1 w/CG)	2 (2 w/CG)	4 (4 w/CG)	4 (3 w/CG)
Unknown	4 (1 w/CG)			
*** Use own (non-VA) device***	7	1	1	5
***Reasons for In-Person***				
Preference/convenience		7	3	
Expedite VVC			4	
Tablet troubleshoot			1	5
Tablet return/”gave up”		1		6
***Reasons for decreased success***				
Connectivity**			8	2
Device issue			1	2
Computer use difficulty			1	3
Physical Impairment**			1	3
Unknown				1

Key

*CG-Caregiver

**Connectivity (WiFi [2], National connectivity issue [4], unknown [4])

**Physical impairment (dexterity [3], visual [1])

### Clinical Pharmacy Assessments

Participants with completed assessments (n = 41) were noted to have a high mean (SD) number of active medications 11 (4.3), with approximately half of the participants involving the caregiver in their medication management (see Table 1). All had medication discrepancies, with a mean 3.8 discrepancies per participant; all of these discrepancies were resolved by the end of the baseline assessment and updated in the medical record. Nearly 1/3 were taking what would be considered potentially inappropriate medications. Of the potentially inappropriate medications identified (n = 15 medications, 1.6 per participant), 4 (25 %) were immediately recommended to decrease or discontinue the medication, 6 (38 %) had a documented reason to continue unchanged; and 6 (38 %) were deferred to the patient’s primary provider.

### Social work assessment and intervention

Of those with completed assessments (*n* = 43), the total mean (SD) number of social work interventions was 1.2 (1.4). Assessments included determination of home safety and need for repair, caregiver health and needs for additional caregiver support, and nutritional and transportation needs, including consideration of others living in the home (such as grandchildren and great-grandchildren). Interventions included referrals to appropriate agencies and resources.

### Short Portable Performance Battery (SPPB) and Actigraphy (activPAL)

As expected given a cohort of participants post hospital discharge with physical therapy needs, baseline SPPB was in the markedly impaired range (mean 4.9) and particularly low in the chair rise test (mean 1.0). Similarly, the mean total number steps per day was low (2334) and the percentage of the day spent in sedentary level activity was high (84 %).

## Discussion

In this disabled post-hospital discharge cohort of older adults with physical therapy goals, a VVC-based assessment and enrollment for a mobility/physical activity intervention program was feasible. The present study makes an important contribution to the literature regarding the feasibility of VVC-assessment of debilitated older adults, some with cognitive impairment and requiring caregiver support, as they enroll in a post-hospitalization intervention.

### Videoconference connectivity issues

Over 2/3 of the participants used VA-supplied tablets. Yet, connectivity/communication concerns were nevertheless common. These concerns were identified as reasons for some Veterans declining participation or disenrolling early. While half of the Veterans were fully successful in VVC (n = 23), 1/3 of these (*n* = 8) and an additional group with at least one failed connection (*n* = 11) requested in-person visits for assistance. One-quarter (*n* = 11) had no VVC success and sought help for tablet troubleshooting, and half of these (*n* = 6) eventually “gave up” trying to connect; difficulty with using the computer and physical impairment (particularly dexterity) were described prominently in this group. On the other hand, Veterans with at least mild cognitive impairment (based on MoCA scores) were present in all connectivity groups and most of these used caregiver support to facilitate VVC. Perhaps surprisingly, there was not a clear difference in VVC success between use of a compatible personal device and the VA-supplied tablet.

From these data we conclude that a substantial proportion of these post-discharge older Veterans need technical support, to include in-person support for many. Yet, VVC seems feasible in those with mild or clinically significant cognitive impairment, assuming the presence of a caregiver. In fact, most of those more recently recruited (1/3 of the entire sample) did not have a caregiver to assist. Some Veterans may refuse to engage in VVC or not ever succeed, even with caregiver support; some caregivers might have also been impaired or had difficulty with using the computer and thus not be able to assist the participant with VVC. Because of the focus on VVC, essentially none of the participants or caregivers used any of the more recent personalized telehealth applications, such as MyHealtheVet, to communicate with the team. This is consistent with our concern, citing nationally representative data in those 65 and older, that use of technology such as email, text messaging or internet is decreased in those with limitations in physical capacity and greater disability, particularly those with vision impairment and memory limitations [18]. Given that these data reflect use from 2017-early 2020, we acknowledge that ease of use of VVC (as well as My HealtheVet) has and continues to advance greatly and that future studies of this impaired cohort may find greater engagement and success in using VVC and other telehealth modalities.

### Modifications for VVC assessment

#### VVC modifications for MoCA

Instead of choosing a more limited MoCA tool (such as the “blind” MoCA), we chose to develop practical, VVC-compatible modifications, such as for the visuospatial/executive portion. The result was that over half of the concurrent, non-medical record MoCA tests (18/32) were done via videoconference. Note that videoconference MoCA administration with analogous modifications has been piloted in patients with mild-severe Alzheimer disease and found to be feasible and reliable in the presence of a caregiver [19]; in the present study, most participants with mild cognitive impairment, and essentially all participants with clinically significant cognitive impairment had caregiver assistance for MoCA assessment. In terms of the effect of VVC modifications on test outcome, only one participant whose MoCA was conducted via VVC lost more than 1 point (scoring a 1 out of 5) on the visuospatial/executive function component. Of further support for the VVC MoCA is that the present study found no mean difference between in-person and VVC scores (although not measured in the same individuals for comparison). Accordingly, home-based “real-world” VVC MoCA administration still needs to be compared further to standard MoCA administration, and may eventually be considered in future required MoCA training and certification.

#### VVC modifications for SPPB

Due to the lack of guidelines to quantitatively assess mobility using a videoconference interface, the SPPB was adapted, particularly in regards to gait speed determination. Mobility testing via videoconference (including VVC) has become common, although to our knowledge, there are few comparisons of face to face versus “real world” home VVC. Video conference based assessments of components of the SPPB, such as the chair rise test, are already being adopted [20]. A key issue is how to handle timed performance, which, assuming a stable WiFi connection with sufficient bandwidth, should be possible, but might require additional cues in the event of a time delay for start and stop times. Note however that the SPPB data from this post-hospital VVC cohort is remarkably similar to a cohort identified to undergo rehabilitation for mobility and physical activity, both inpatient and outpatient via telehealth devices, and whose SPPB was evaluated in person [21]. Note also that participants were enrolling in an intervention, thus serving as their own control, and VVC assessment modifications were planned to be continued throughout the 6 month follow-up.

### Need for VVC data in older adults: comorbidities, cognitive impairment, and caregiver participation

In recent surveys, only 1/3 of VA Telehealth tablet users were over age 65, and comorbid Veterans, i.e. those with ≥7 chronic conditions, were less likely to use their tablets [22]. Recently, of 118 older Veterans (mean age 73) appointed to outpatient visits during COVID-19, only 63 (53 %) were willing and able to participate in a VVC appointment; of note, 30 (26 %) had cognitive impairment or dementia [23]. Of the 35 VVC appointments scheduled, 27/35 (77 %) were successfully completed but 13 of these 27 (48 %) received support from a caregiver. These data are consistent with the findings of the present study. Further studies are needed to determine best practices to optimize VVC for those with comorbidities, and particularly those with cognitive impairment, and how to leverage caregiver participation.

### Extent of cohort impairment

As expected, this post-hospital discharge cohort of older adults with rehabilitation needs had evidence of ADL disability and mobility impairments. Mobility (SPPB) performance (the chair rise scores in particular) and the number of steps per day were in the markedly low/impaired range, while the percent time spent in sedentary activity was high. These results are similar to those in rehabilitation patients provided with telehealth modalities post-hospital discharge [21].

In terms of other assessments at baseline, consistent with a medically complex cohort was the high number of medications, and despite the help of a caregiver in at about ½ of the participants, medication discrepancies and inappropriate medications were still found. Not surprisingly, the social complexity was equally high with the need for complex social work interventions.

### Telehealth for exercise and rehabilitation

Use of telehealth for exercise in specific diseases (such as cancer) is well-accepted and has been adapted to a number of modalities, including web-based, mobile applications, text messaging, and telephone interventions [24]. Video telehealth rehabilitation services, particularly physical therapy, are growing, although there are few studies that include older adults over age 80 [25]. These programs may also include other interventions beyond exercise that may help to improve rehabilitation outcomes. For example, video-based telehealth pulmonary rehabilitation for COPD reduced 30-day rehospitalization and included educational content in addition to the exercise instruction [26]. Interventions which lead to increased physical activity and reduced sedentary behavior use many of these same telehealth modalities, but tend to exclude older adults of more advanced age (e.g. >70 years), multiple comorbidities (versus single diseases such as diabetes mellitus), and those with cognitive impairment and/or who require caregiver support [10].

### Strengths

A major strength is the targeting of an older, recently hospitalized, disabled cohort with rehabilitation needs, and who might benefit from a videoconference-based intervention. The inclusion of cognitively impaired individuals, using a caregiver support model, is also important to consider. Integrating this important cohort with disability and rehabilitation potential with a videoconference assessment and intervention seems critical to larger uptake of the VVC model.

#### Limitations

A few features of this pilot study changed over time, including the requirement of a caregiver and the amount of in-person assessment utilized. With careful instruction, the actigraphy placement was eventually completed remotely and still provided valid data. Given the relative novelty of the program and our relative inexperience with videoconference versions of parts of the assessment, these changes were probably inevitable. These have been carefully documented and noted above and thought not to result in a systematic bias of the results.

##### Delay in enrollment, assessment, and rehabilitation

Completion of enrollment and consent was delayed, completed usually 1–3 weeks post-charge. Baseline assessments were completed within 1–2 weeks of completed enrollment. While the protocol was initially designed to recruit participants prior to discharge, discharge plan variability, inconsistent contact with the inpatient, and patient preference to return home prior to consent required other recruitment approaches. These approaches included a telephone call, meeting at a follow-up outpatient appointment, or an introduction letter with team member phone follow-up. These might be expected in this functionally impaired, medically and socially complex cohort. An effect of the delay of functional outcomes at baseline is unlikely given there may also have been a delay in the participant receiving outpatient or home-based therapy.

## Conclusions

Disabled older post-hospital discharged Veterans with physical therapy goals can be VA Video Connect (VVC) assessed and enrolled into a mobility/physical activity intervention. While 2/3 used the VA-supplied tablets, a substantial proportion required technical support, including in-person support for many. Yet, VVC seems feasible in those with mild or clinically significant cognitive impairment, assuming the presence of an able caregiver. Modifications of assessment tools were needed for the VVC interface, and while appearing feasible, will require further study.

## Data Availability

The datasets used and/or analyzed during the current study are available from the corresponding author on reasonable request.
